# Intra-Testicular Signals Regulate Germ Cell Progression and Production of Qualitatively Mature Spermatozoa in Vertebrates

**DOI:** 10.3389/fendo.2014.00069

**Published:** 2014-05-08

**Authors:** Rosaria Meccariello, Rosanna Chianese, Teresa Chioccarelli, Vincenza Ciaramella, Silvia Fasano, Riccardo Pierantoni, Gilda Cobellis

**Affiliations:** ^1^Dipartimento di Scienze Motorie e del Benessere, Università di Napoli Parthenope, Naples, Italy; ^2^Dipartimento di Medicina Sperimentale sez “F. Bottazzi”, Seconda Università degli Studi di Napoli, Naples, Italy

**Keywords:** testis, spermatogenesis, GnRH, kisspeptins, estrogens, sperm quality, spermatozoa

## Abstract

Spermatogenesis, a highly conserved process in vertebrates, is mainly under the hypothalamic–pituitary control, being regulated by the secretion of pituitary gonadotropins, follicle stimulating hormone, and luteinizing hormone, in response to stimulation exerted by gonadotropin releasing hormone from hypothalamic neurons. At testicular level, gonadotropins bind specific receptors located on the somatic cells regulating the production of steroids and factors necessary to ensure a correct spermatogenesis. Indeed, besides the endocrine route, a complex network of cell-to-cell communications regulates germ cell progression, and a combination of endocrine and intra-gonadal signals sustains the production of high quality mature spermatozoa. In this review, we focus on the recent advances in the area of the intra-gonadal signals supporting sperm development.

## Introduction

In vertebrates, spermatogenesis is a hormonally controlled mechanism charged to produce gametes useful for reproduction. The production of high standard quality gametes is the main goal to preserve reproduction.

Spermatogenesis develops as a process consisting of mitotic, meiotic, and differentiation steps promoting germ cell progression from spermatogonia-to-spermatozoa (SPZ). In male, the hypothalamus–pituitary–gonadal axis supports germ cell progression, via gonadotropin releasing hormone (GnRH)–gonadotropin–steroid production and its activity is finely regulated by positive and negative feedbacks. Furthermore, a network of intra-gonadal factors, organized in a complex stage-specific multi-factorial net, is responsible for spermatogenesis control (1).

Using a comparative approach, this review summarizes the intriguing and sometimes conflicting information about the intra-testicular role played by GnRH, Kisspeptin, and estrogens in germ cell progression and production of high standard quality sperm.

## GnRH, a Historical Modulator of Testis Physiology

The GnRH, crucial player of the neural control of vertebrate reproduction, was originally isolated from the hypothalamus of pig and sheep (2). Basically, GnRH stimulates the synthesis and the discharge of pituitary gonadotropins [follicle stimulating hormone and luteinizing hormone (FSH and LH), respectively], which in turn induce both gametogenesis and the production of gonadal steroids. At present, 25 GnRH forms have been identified in protochordates and vertebrates (3, 4) and in many vertebrates three GnRH molecular forms have been identified: GnRH-1, GnRH-2, and GnRH-3 (formerly known as mammalian, chicken-II, and salmon GnRH, respectively) (3). GnRH action is mediated through high-affinity binding with the GnRH receptor (GnRH-R) (5, 6), a rhodopsin-like seven trans-membrane G protein-coupled receptor (GPCR). In vertebrates, GnRH-Rs exhibit a wide range of subtypes and alternate splicing derived forms (1, 3, 5–7). The presence of multiple forms of GnRHs and GnRH-Rs in the brain, with specific expression profiles, suggests the existence of different functional roles: in fact, GnRH-1 is considered the final regulator of the pituitary–gonadal axis; GnRH-2 is supposed to play a function for the control of sexual behavior, food intake, energy balance, stress, and many other environmental cues; GnRH-3, found only in the telencephalon of teleost fish, probably acts as neuro-modulator (1, 3, 8).

Extrahypothalamic synthesis and function of GnRHs and GnRH-Rs have been detected in many reproductive tissues in vertebrates, including human (gonads, prostate, endometrial tissue, oviduct, placenta), and in cancer cells (1, 5, 9–11).

GnRH plays several conserved roles in testis physiology, being the main paracrine modulator of the Leydig–Sertoli, Sertoli–germ cell, Sertoli–peritubular cell communications (1, 12). In this context, it drives steroidogenesis, germ cells progression, and acquisition of SPZ functions (1, 12–15).

The demonstration of a direct GnRH effect on testis has been provided in fish, frog, rodent, and human Leydig cells showing GnRH-specific high-affinity binding sites (1, 3, 15, 16). The finding of *GnRH* mRNA in Sertoli and spermatogenic cells in different species (17) suggests its involvement in paracrine Leydig–Sertoli cell communication (12). A similar pattern of expression has been confirmed in human (17), expressing two GnRH molecular forms and two GnRH-Rs (18, 19). However, the identification of *GnRH-R2* antisense transcript in human testis (20) and the presence of frame-shift mutations and stop codons in human *GnRH-R2* (5) may indicate that these transcripts are not really functional.

The major reported effect of GnRH on vertebrate testis physiology concerns the modulation of steroidogenesis in *in vivo* and *in vitro* systems (1, 21–23). Interestingly, in elasmobranch and in dipnoi, this effect appears to be exerted trough the endocrine route (24, 25). Both GnRH-1 and GnRH-2 agonists have the ability to stimulate mouse pre-pubertal Leydig cell steroidogenesis, in a dose- and time-dependent manner, via transcriptional activation of 3β-hydroxy-steroid dehydrogenase (3β-HSD) (23). Accordingly, in human, the expression levels of *GnRH-1*, *GnRH-2*, *GnRH-Rs*, *cytochrome P450 side-chain cleavage* (*CYP11A1*), *3β-HSD type 2* enzyme, and the intra-testicular testosterone (T) levels are significantly increased in patients with spermatogenic failure (26). At molecular level, the transduction pathway involving the GnRH agonist-dependent activation of ERK1/2 has been reported (27). Interestingly, in mouse testis, GnRH-R activity well correlates with the increased steroidogenic activity observed during pubertal and adult stages and its decline parallels the decreased steroidogenic activity observed during the senescence (28). These expression profiles are consistent with the increasing expression of the gonadotropin inhibitory hormone (GnIH) during senescence, providing evidences of local interaction between GnRH and GnIH. The testicular localization of GnIH and its receptor GPR147, in both mammals and birds, opens new perspectives in the autocrine/paracrine control of testicular activity suggesting a possible interplay between GnRH and GnIH in order to modulate T secretion and spermatogenesis (29). Furthermore, GnRH activity in Leydig cells is not restricted to T production but is extended to the development of rat progenitor Leydig cells both *in vivo* and *in vitro* (30).

Several studies, carried out in cancer cell lines, demonstrated a direct anti-proliferative/apoptotic effect of GnRH and its synthetic agonists (31, 32). Accordingly, GnRH activity is a well-known modulator of germ cell apoptosis during the regression of fish gonad (33, 34). In rodents, GnRH agonists stimulate spermatogenic colony formation following spermatogonia (SPG) transplantation (35, 36) and induce SPG proliferation in damaged testis (37). In mollusk, a scallop GnRH-like peptide stimulates SPG cell division (38). In amphibian, a GnRH agonist induces G1-S transition of SPG cell cycle (39–43) whereas, in mouse, GnRH is expressed in gonocytes at birth (28). At molecular level, in the anuran amphibian *Rana esculenta*, SPG proliferation requires the cooperation between estradiol (E_2_) and GnRH, in a mechanism involving the E_2_-dependent transcriptional activation of *c-fos* (42) and a GnRH-mediated translocation of FOS protein from the SPG cytoplasm into the nucleus (43). Thus, GnRH activity may represent a key controller of proliferative/anti-proliferative events characteristic of testis renewal. Consistently, it has been found that GnRH induces proliferation of partially differentiated gonadotrope cells (44).

Lastly, the ability of GnRH agonists to induce spermiation (45) and the localization of GnRH and/or GnRH-Rs in spermatids (SPTs) and SPZ in mammalian and non-mammalian vertebrates (17, 28, 46, 47) suggest the involvement of GnRH signaling in SPZ functions and fertilization. Accordingly, GnRH antagonists inhibit, *in vivo* and *in vitro*, fertilization in rodents (14) whereas sperm binding to the human zona pellucida and calcium influx in response to GnRH and progesterone have been reported (13), providing evidence of functional role of GnRH-Rs in human SPZ.

**Figure 1:**
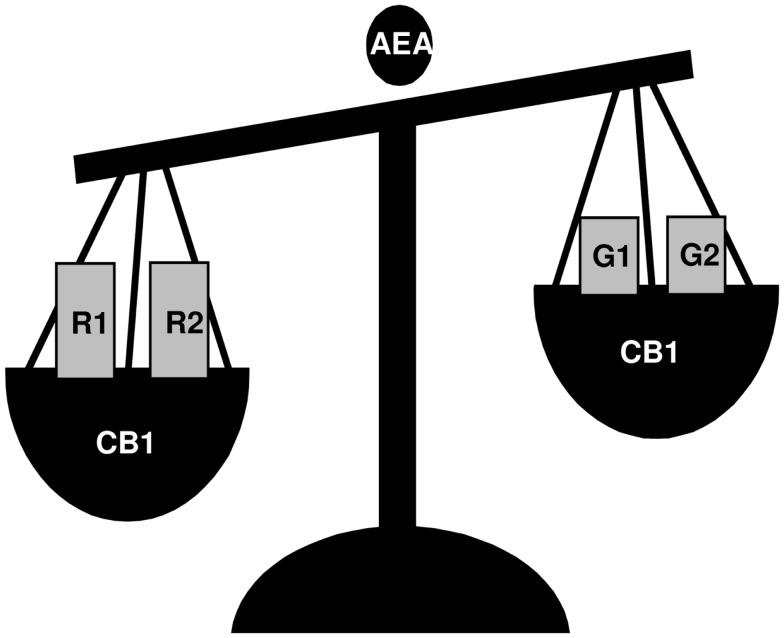
**A schematic view of the effects of AEA incubation on *GnRH-1* (G1), *GnRH-2* (G2), *GnRH-R1* (R1), and *GnRH-R2* (R2) expression in frog *R. esculenta* diencephalon**. Animals were collected in June and testes were incubated *in vitro* for 1 h. Via CB1 activation, AEA treatment significantly increased the expression of *GnRH-R1* and *GnRH-R2* whereas it decreased the expression of both *GnRH-1* and *GnRH-2*; no effect on *GnRH-R3* was observed.

**Figure 2:**
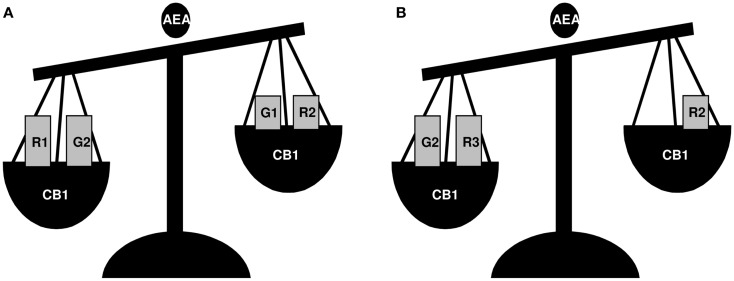
**A schematic view of the effects of AEA treatment on *GnRH-1* (G1), *GnRH-2* (G2), *GnRH-R1* (R1), *GnRH-R2* (R2), and *GnRH-R3* (R3) expression in frog *R. esculenta* testis**. Animals were collected in June **(A)** and February **(B)** and testes were incubated *in vitro* for 1 h. In June, AEA treatment significantly increased the expression of *GnRH-R1* and *GnRH-2* whereas it decreased those of *GnRH-1* and *GnRH-R2*, and had no effect on *GnRH-R3*; in February, AEA treatment increased *GnRH-2* and *GnRH-R3* expression, decreased *GnRH-R2*, and had no effect on *GnRH-1* or *GnRH-R1*. In both periods, AEA-dependent effects occurred via CB1 activation.

The above indicated intra-testicular activity of GnRH has been described in detail in the frog *R. esculenta*, a species showing a complex GnRH system, deeply characterized at testicular level (46). In this seasonal breeder, two GnRH molecular forms (GnRH-1 and GnRH-2) and three receptor forms (GnRH-R1, -R2, -R3) (48) with specific expression pattern and localization in testis during the annual reproductive cycle (46) have been identified. *In situ* hybridization suggests a different role for *GnRH-1* and *GnRH-2*, as *GnRH-1* and *GnRH-R1* seem to be linked to germ cell progression and interstitial compartment activity, whereas *GnRH-2* and *GnRH-R2* seem to be linked to sperm function and release (46), confirming the hypothesis that each ligand might be involved in the modulation of specific processes. Interestingly, this functional portioning well correlates with the differential modulation of GnRH system counterparts exerted via the activation of endocannabinoid system, an evolutionarily conserved system deeply involved in central and local control of reproductive functions (49–52). At central level, in mammals, endocannabinoids interfere with GnRH production (53, 54) and signaling (55). In frog diencephalons, they modulate the expression of *GnRH-1*/*GnRH-2* (48, 56, 57) – both hypophysiotropic factors (1), *GnRH-R1* and *GnRH-R2* (48) (Figure 1). Furthermore, in frog testis, the endocannabinoid anandamide (AEA), via type 1 cannabinoid receptor (CB1) activation, modulates testicular GnRH activity at multiple levels and in a stage-dependent manner (46) (Figure 2). Interestingly, the activation of cannabinoid receptors other than CB1, such as the vanilloid transient receptor type 1 (TRPV1), differentially modulates the expression level of *GnRHs*/*GnRH-Rs*, but in an opposite manner as compared with CB1 (58). Thus, the transcriptional switch on/off of testicular GnRH system is finely toned through the activation of specific endocannabinoid receptors, providing evidence of a central role of this system in the local modulation of GnRH activity.

## Kisspeptins, Possible Players in Testis Physiology Currently Under Investigation

Kisspeptins are a novel class of neuro-peptides with a key position in the scenario of reproduction. They are encoded by the *kiss1* gene, originally discovered as a metastasis-suppressor gene in 1996 (59), and they are initially produced as an unstable 145-amino acid precursor peptide (kp145), then cleaved into shorter peptides (kp-54, kp14, kp-13, and kp-10). Interestingly, all kisspeptin shorter peptides are biologically active due to the binding to the “kiss” receptor GPR54 (60). The primary targets of kisspeptins are just the hypothalamic GnRH-secreting neurons (61) and, similarly to the deletion/mutation of *GnRH* or *GnRH-R* genes, target disruption of both *kiss1* and *GPR54* leads to hypogonadotropic hypogonadism and lack of sexual maturation (62, 63). Accordingly, the administration of kisspeptins accelerates the timing of puberty onset in fish (64–67) and mammals (68, 69), whereas circulating higher kisspeptin levels have been observed in clinical cases of precocious puberty in human (70, 71).

Several studies have been focused on the characterization of the kisspeptin-dependent signaling in the hypothalamus, with particular concern to the negative and the positive feedback action of sex steroids on *kiss1* gene expression in the arcuate and the antero-ventral-preoptic nucleus, respectively [for review see Ref. (72)]. Therefore, in the last years, the idea that kisspeptin signaling is an essential guardian angel of reproduction, through the regulation of GnRH neurons, took place. These views strongly stride with evidences that genetic ablation of nearly all kisspeptin neurons does not impair reproduction, suggesting that possible compensatory mechanisms rescue reproduction (73). Probably, kisspeptin neurons and related products are in excess of what is really required to support reproductive functions. In this respect, male and female mice with a 95% reduction in *kiss1* transcript levels are normal and sub-fertile, respectively. This suggests that an overproduction of kisspeptin represents a failsafe to guarantee reproductive success (74).

A novel chapter of kisspeptin saga concerns the possible intra-gonadal action of these molecules. Kiss1 and/or GPR54 have been observed in several peripheral tissues, gonads included. In particular, the presence of both ligand and receptor has been observed in the human placenta (75) and testis (60, 75) whereas *GPR54* alone has been detected in mouse (76), rat (77), rhesus monkey (78, 79), and frog (80) testis. However, the functional mechanisms of kisspeptin/GPR54 system in gonads remain to be elucidated and several conflicting data concerning the direct involvement of kisspeptin activity in testis physiology emerged.

Long term kisspeptin-10 (kp-10) (81) and/or kp-54 (82) administration in maturing and adult rat testes gives rise to degenerative effects on spermatogenesis and suppresses the circulating levels of LH and T; no effects have been registered upon FSH levels. Specifically, germ cell number significantly decreases, many germ cells appear regressed, atrophied, and in necrosis; round and elongated SPTs show abnormal acrosome; intraepithelial vacuolization is visible, interstitial spaces are enlarged, and the germinal epithelium is irregularly shaped. Leydig cells frequently lose contacts with the seminiferous tubules and Sertoli–germ cell interaction is destroyed (81). A similar degenerative effect – caused by continuous administration of kp-10 – has also been discovered in rat seminal vesicles (83) and pre-pubertal prostate gland (84). Conversely, a physiological role of kisspeptins in testis has been completely excluded in mouse (85) and conflicting data concerning the localization of kiss1/GPR54 protein and mRNA recently emerged. The use of different antisera, strategies, and strains as well might be taken in account to explain these discrepancies and the missing overlapping in mRNA/protein detection described so far. In fact, in transgenic mice with LacZ targeted to either *kiss1* or *GPR54* genes, *kiss1* and *GPR54* mRNA have been localized in mouse round SPTs, whereas kisspeptin protein has been shown in Leydig cells, with no staining in SPTs (85). Conversely, both GPR54 and kiss1 immunoreactivity has been provided in both Leydig and germ cells (primordial germ cells and elongating SPTs) with significant age-related variations (28). Studies conducted in Leydig cell line MA-10 – a cell line that expresses LH receptors and responds to human chorionic gonadotropin (hCG) stimulation, producing progesterone as major steroid hormone – confirm that these cells produced *GPR54* mRNA, but were unable to show any *kiss1* expression (85). Despite *GPR54* expression, from a functional point of view kp-10 does not exert any significant direct effects on steroid production in both MA-10 cell line or in physiological systems, such as mouse seminiferous tubule explants (85). However, evidences reported in other species examined so far, pointed out a possible role of kisspeptin system just in steroidogenetic activity. Although Leydig cells do not show any kisspeptin and/or GPR54 immuno-localization in rhesus monkey (78), intra-testicular action on steroidogenesis (79) has been demonstrated in monkeys treated with acyline, a GnRH-R antagonist (86), just to exclusively investigate kisspeptin activity without any influence of pituitary gonadotropic drive. In these clamped monkeys, kp-10 has a synergic effect with hCG to induce T production (79). Anyway, the real possible mechanism through which kisspeptin enhances T production in primates is not clear and may require additional paracrine routes involved in Leydig–Sertoli cell communications. In fact, in rhesus monkey kisspeptin immunoreactivity has been detected in spermatocytes (SPCs) and SPTs, whereas GPR54 has been localized in SPCs and Sertoli cells (78). Thus kisspeptin – produced by germ cells – might act in an autocrine/paracrine manner to control the progression of the spermatogenesis and/or to modulate Sertoli cells activity. It is noteworthy, however, that intravenous injection of the kisspeptin antagonist 234 (kp-234) (87) does not alter plasma T levels in adult rhesus monkey. Interestingly, Anjum and co-workers reported that kisspeptin expression – analyzed by slot blot analysis in Leydig cells of Parkes strain mice – significantly decreases from birth to pre-pubertal testis, increases during pubertal period, decreases in reproductive active mouse to further increase during the senescence. These expression profiles well correlate to GnIH expression and to the decreased steroidogenic activity observed during the senescence, providing evidence of a possible involvement of kisspeptin in the control of steroidogenesis in cooperation with testicular GnIH (28).

The detection of *kiss1* and *GPR54* mRNA in round/elongating SPTs (28, 78, 85) raises the possibility that autocrine or paracrine kisspeptin actions might be involved in spermiogenesis and in the acquisition of sperm functions, as recently demonstrated in human SPZ by Candenas and co-workers (88). This group immunolocalized kisspeptin and GPR54 in the post-acrosome region of the human SPZ head and in the equatorial segment of the tail, providing also evidence of some regulatory actions. In fact, 1 μM kp-13 increases [Ca^2+^]_i_ and induces a small, but significant change in sperm motility, leading to motility trajectories that characterize hyper-activated SPZ. Instead, the same treatment has no effect on acrosome reaction (88). Very recently, in mouse, GPR54 has been specifically localized in the acrosomal region of SPTs and mature SPZ whereas kisspeptin expression has been detected in the cumulus–oocyte complex and oviductal epithelium of ovarian and oviductal tissue (89). Since SPZ treatment with kp-234 decreases the *in vitro* fertilization rates, evidence emerged that kisspeptin modulates fertilization capability in mammals (89).

Interestingly, in sexually immature scombroid fish, kp-15 peripheral administration induced spermiation (67), accordingly to *GPR54* expression detected in the myoid peritubular cells in amphibians (80), indicating a possible involvement in sperm transport and release.

**Figure 3:**
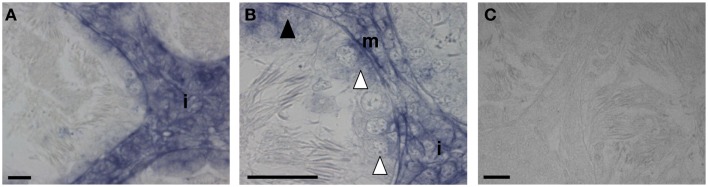
**Sections of *R. esculenta* testis, collected in February, analyzed by *in situ* hybridization for *GPR54***. *GPR54* mRNA was detected in the interstitial compartment **(A,B)**, in primary spermatogonia **(B)**, in secondary spermatogonia cysts **(B)** as well as in myoid peritubular cells **(B)**. The specificity of signals was tested through the reaction with a sense riboprobe **(C)**. i, Interstitial compartment; white arrow head, ISPG; dark arrow head, IISPG; m, myoid peritubular cells; scale bar: 20 μm.

Compelling evidence about gonadal activity of kisspeptin system recently comes from a non-mammalian vertebrate, the anuran amphibian, the frog *R. esculenta*. In this seasonal breeder, germ cell progression is under the control of endocrine, environmental, and gonadal factors (90, 91), whereas spermatogenesis proceeds in cysts, typical formations consisting of Sertoli cells enveloping cluster of germ cells at a synchronous stage (91). During the frog annual sexual cycle, *GPR54* mRNA has been analyzed in testis, showing higher expression at the end of the winter stasis and during the breeding season (80). In these periods, in an E_2_-dependent fashion, the recruitment of SPG and the onset of a new spermatogenetic wave take place (42, 91, 92). Consistently, in February, *GPR54* mRNA has been revealed in primary and secondary SPG by *in situ* hybridization (Figure 3) (80) accordingly to kisspeptin localization in primordial germ cells observed in mouse (28). In proliferating germ cells, a strong expression of *GPR54* mRNA has been found in interstitial compartment of frog testis all over the annual sexual cycle (Figure 3). Contrary to human, in frog post meiotic cells and SPZ do not express *GPR54* mRNA, but it is not excluded that the *GPR54* mRNA produced in SPG might be translated in later stages. Since E_2_ is likely to be involved in various aspects of testicular activity such as steroidogenesis and primary SPG proliferation (42, 93–95), a possible relationship between E_2_ and kisspeptin/GPR54 has been analyzed in frogs. In this respect, an E_2_-dependent modulation of *GPR54* expression has been reported in testis. In addition, kp-10, *in vitro*, is able to modulate both *GPR54* and *ER*α expression at the end of the winter stasis (February) as well as during the breeding season (March) (80). Therefore, via kisspeptins/GPR54 activation, E_2_ might regulate steroidogenic activity and SPG proliferation. This hypothesis is supported by the localization of *GPR54* mRNA that well correlates with the sites of E_2_ action occurring in frog testis (90). Thus, the expression of *GPR54* inside the interstitium and in proliferating SPG, as well as its E_2_-dependent expression, strongly support the hypothesis that kisspeptin might have a direct involvement in the onset of the spermatogenetic wave. Accordingly, subcutaneous administration of kp-15 accelerates spermatogenesis in the pre-pubertal teleost *Scomber japonicus* without any significant change in the expression of hypothalamic *GnRH-1* and pituitary *FSHβ* and *LHβ* subunits (66). In addition, kp-10 involvement in differentiation events has been further confirmed in the rhesus monkey derived stem cell line r366.4 (96).

It is evident that the several controversies regarding the “kisspeptin saga” make their history more intriguing with many “behind-the-scenes” yet to be written.

## Estrogens and Sperm Quality

Traditionally, E_2_ is stereotyped as the “female” and T as the “male” hormone. E_2_ and T are instead present in both males and females, and in male the ratio between the two hormones controls reproduction via specific receptors (16). To date, nuclear (ERα and ERβ) and membrane-bound (GPR30) receptors, able to respond to E_2_ via genomic and non-genomic pathways, respectively, have been identified [for review see Ref. (97, 98)].

Estrogens are synthesized via the irreversible transformation of androgens by the aromatase (P450arom; *Cyp19A1* is the related gene), an enzyme expressed in the endoplasmic reticulum of testicular cells. In male, E_2_ is indeed primarily synthesized in the testis, which expresses also the specific receptors, ERα and ERβ (16). Recently, GPR30 has been studied in fish, rat, and human and localized in somatic (rat and human) and germ (fish and rat) cells (99–102).

P450arom and ERs expression has been studied in mammalian and non-mammalian testis and the specific mRNA and/or proteins localized in the interstitial (Leydig cells) and tubular (Sertoli and germ cells) compartments, depending on the species [for reviews see Ref. (1, 16, 97, 103)], demonstrating that both somatic and germ cells are able to produce E_2_ that can act locally.

In vertebrates, E_2_ acts at both central (hypothalamus and hypophysis) (55, 104) and local (testis, efferent ductules, and epididymis) (1, 105, 106) levels and studies in mammalian and non-mammalian species show that E_2_ regulates proliferation (gonocytes, SPG, Leydig cells), apoptosis (pachytene SPC, Sertoli cells), and differentiation (SPTs) of germ and somatic cells, as well as it regulates spermiation, transport and motility of SPZ, epididymal sperm maturation, and scrotal testicular descent (42, 43, 80, 97, 107–116). Some of these functions are evolutionarily conserved from fish to mammals demonstrating that E_2_ plays an important role in male reproduction physiology in vertebrates (1, 90, 117). Expression profiling of spermatogenesis in the rainbow trout identifies evolutionarily conserved genes involved in male gonadal maturation (118). Accordingly, E_2_-responsive genes have been characterized in gonads enriched of SPG or in isolated germ cells: in both frog (42, 108) and fish (118, 119), some of these genes are associated to proliferation.

To date, although tissue and cell culture experiments show that E_2_ may act on germ cells, its direct effect in *in vivo* systems has not yet been fully elucidated. However, data obtained in mouse, rat, and human models clearly show that E_2_ is important to produce and sustain high standard quality mature SPZ. Two main observations suggest that E_2_ is able to act locally into the testis: (1) germ cells express both P450arom and ER, in particular SPTs (120) produce E_2_ that may act via specific receptors (121); (2) Sertoli cell barrier envelops the germinal epithelium, from SPCs to SPTs/SPZ, ensuring a specific micro-environment that allows a correct germ cell progression.

In mouse, P450arom activity is high in germ cells and in particular in SPTs, while is lowered in the interstitial compartment (120). Among germ cells, mainly SPTs and SPZ are responsive to inhibition/inactivation of P450arom and to low E_2_ levels. Early studies, demonstrated that when rat (122, 123) or bonnet monkey (115) were treated with aromatase inhibitors, degeneration of round SPT and a massive decrease of elongated SPTs was found. Later, D’Souza showed that round SPT differentiation (steps 1–6) was largely dependent on E_2_, whereas SPT elongation (steps 8–19) was androgen dependent (124). Indeed, high intra-testicular E_2_ levels preserve round SPTs (steps 1–6) whereas T deficiency, induced by E_2_, originate pyknotic bodies in elongated/condensed SPTs (steps 8–19) (124). Consistently, loss of E_2_ in human testis promotes apoptosis of round SPTs with loss of elongated SPTs (125) and viable SPZ (126). Therefore, E_2_ is now considered as a survival factor for SPTs and SPZ.

The bulk of information about the role of E_2_ in germ cell differentiation, from SPT-to-SPZ, came from studies on mutant mice such as the hypogonadic (*hpg*), the *Cyp19A1* knock-out (ArKO), and the *Cb1* knock-out (*Cb1*^−/−^) (55, 127, 128).

Due to a natural *GnRH* gene deletion, the *hpg* mice are functionally deficient in gonadotropins and sex steroids and show meiosis arrest at pachytene stage. Treatment with E_2_ or ERα agonists restored meiosis in these animals which, in absence of T, produce haploid elongated SPTs (129). The E_2_ treatment alone was as effective as FSH alone and the combination of both hormones did not produce a greater effect (130). Authors concluded that E_2_ likely acts on *hpg* testis via a mechanism involving a weak neuroendocrine activation of FSH secretion (128–130).

The phenotype of ArKO mice and experimental analysis carried out using this mutant mice counteract with this conclusion. ArKO males (127) are initially fertile, but they develop progressive infertility between 4.5 months and 1 year. In the SPTs of these animals, multiple acrosome vesicles, irregularly scattered over the nuclear surface, are observed (127) suggesting that acrosome biogenesis may be an E_2_-dependent process. Accordingly, P450arom is at high levels in the Golgi complex of developing SPT (120). In ArKO mice, spermatogenesis is primarily arrested at early stages, with a decrease of round and elongated SPT numbers, without any detectable change of circulating FSH levels (127). Dietary phytoestrogens may partially prevent disruption of ArKO mice spermatogenesis, avoiding the decline of germ cell number. Interestingly, when young ArKO mice were exposed to a phytoestrogen free diet, the phenotype was severely disrupted as compared with mice under normal diet. This occurred in absence of a decreased gonadotropic stimulus, suggesting that the effects of dietary phytoestrogens are independent of changes concerning the pituitary–gonadal axis and they are probably related to direct activation of testicular ERs (131). In agreement with this conclusion, E_2_ administration in irradiated rats suppressed serum LH, FSH, and T (both plasma and intratesticular) levels (132) and produced the recovery of spermatogenesis (133, 134) suggesting a gonadotropin-independent E_2_ activity. However, gynecomastia and cardiovascular problems are secondary effects related to E_2_ treatment and represent the major impediment to clinical application. Recently, it has been suggested that the phytoestrogen genistein may be a true substitute for E_2_ (135).

Concerning the *Cb1*^−/−^ mouse, it is a genetically modified animal model showing *Cb1*-gene deletion (136). This gene codifies CB1, which is broadly expressed in hypothalamus, pituitary, and testis (137, 138) of many vertebrates, from fish to mammals [for review see Ref. (52)]. CB1 is involved in GnRH and gonadotropin production (55–57, 139–141) at testicular level, it regulates both spermatogenesis (15, 46, 58, 137, 138, 142–145) and steroidogenesis (146, 147). Interestingly, *Cb1*^−/−^ mice exhibit endocrine features in common with *hpg* and ArKO models: (1) down regulation of *GnRH* and *GnRH*-*R* mRNA, (2) low LH release and low expression of *FSHβ* mRNA, (3) low T production, and (4) low E_2_ plasma levels. Morphological and molecular analyses of epididymis and 3β-HSD, which are responsive to T, suggest that even low, T levels are enough (55). Unlike *hpg* and ArKO mice, *Cb1*^−/−^ mutants are fertile; they show a quantitatively normal production of SPZ although, similarly to some fertile men, a consistent aliquot shows abnormalities (148, 149) that are mainly related to the motility and to chromatin quality (histone content, chromatin packaging, DNA integrity, and nuclear size, useful parameters to classify sperm chromatin quality). Therefore, *Cb1*^−/−^ mice exhibit endocrine and phenotypic features, which are useful to extend the above studies about the role of E_2_ in SPT differentiation and in the maintenance of sperm quality. Interestingly, when *Cb1*^−/−^ mice were treated with E_2_, all the abovementioned chromatin quality indices improved in SPZ (55, 150). Therefore, sperm chromatin quality appears to be responsive to E_2_ treatment (151). Interestingly, *ERα* and *ERβ* polymorphisms have been associated with semen quality (152). Accordingly, P450arom, either mRNA or protein, has been proposed as marker of sperm quality in men. Indeed, Carreau and co-workers reported that, in human ejaculated SPZ, the immotile sperm fraction showed low levels of P450arom, both mRNA and protein activity (30 and 50%, respectively), as compared with the motile sperm fraction (153–155). In addition, the same authors have recently reported that in SPZ from asthenospermic, teratospermic, and asthenoteratospermic patients, *P450arom* mRNA levels were progressively lower as compared with SPZ from control patients (156). The hypothesis that E_2_ treatment improves motility by enhancing oxidative metabolism and the intracellular ATP concentrations in human sperm (157, 158) well fit with the observation that E_2_ can regulate mitochondrial function in MCF7 cells by increasing nuclear respiratory factor-1 expression (159). However, in mouse, E_2_ and phytoestrogens are able to improve capacitation as well as acrosome reaction and fertilizing capacity of SPZ (160), while natural and synthetic estrogens have stimulatory effect on boar sperm capacitation *in vitro* (161).

Results from mutant animal models, here reported, in combination with case reports concerning patients with few testicular germ cells or decreased sperm motility and number, have a common root: they are characterized by E_2_ deficiency due to the mutation or low expression of *Cyp19A1* gene ((126, 162–164), suggesting that E_2_ may have a instrumental role in quality sperm and its action is much more complex than previously predicted or suggested by ERα knock-out mice, which show impaired fluid re-adsorption within the efferent ducts as cause of sterility (105).

## Closing Remarks

In the last years, data provided by literature evidence that, besides endocrine route, intra-testicular paracrine and autocrine communications are fundamental to sustain spermatogenesis in order to gain high standard quality SPZ. New roles for stereotyped hypothalamic and female hormones – GnRH and E_2_, respectively emerged, new potential modulators such as kisspeptins have been identified as well, but conflicting data reveal that several issues need to be further investigated. The modulators here reported – GnRH, kisspeptin, and estrogens – are critical for a successful spermatogenesis as clearly demonstrated by clinical cases of infertility in humans. However, several questions are still open. These different modulators strongly cooperate at hypothalamic level whereas, at testicular level, they control similar events (Leydig cell functions, proliferation/differentiation events, sperm functions); conversely, their possible local crosstalk is far away to be elucidated. Similarly, they may trigger, independently from each other, pathways controlling the same aspects that might represent two sides of the same coin. Both a comparative approach and the use of genetically modified experimental models may represent a successful tool to make giant strides in the building of general models, but to extricate this intriguing story, there is still much to be done.

## Conflict of Interest Statement

The authors declare that the research was conducted in the absence of any commercial or financial relationships that could be construed as a potential conflict of interest.
